# Comparative Analysis of Artificial Intelligence Models for Accurate Estimation of Groundwater Nitrate Concentration

**DOI:** 10.3390/s20205763

**Published:** 2020-10-12

**Authors:** Shahab S. Band, Saeid Janizadeh, Subodh Chandra Pal, Indrajit Chowdhuri, Zhaleh Siabi, Akbar Norouzi, Assefa M. Melesse, Manouchehr Shokri, Amirhosein Mosavi

**Affiliations:** 1 Institute of Research and Development, Duy Tan University, Da Nang 550000, Vietnam; 2 Future Technology Research Center, National Yunlin University of Science and Technology, 123 University Road, Section 3, Douliou, Yunlin 64002, Taiwan; 3 Department of Watershed Management Engineering and Sciences, Faculty in Natural Resources and Marine Science, Tarbiat Modares University, Tehran 14115-111, Iran; janizadehsaeid@modares.ac.ir; 4 Department of Geography, The University of Burdwan, West Bengal, Burdwan 713104, India; scpal@geo.buruniv.ac.in (S.C.P.); indrajitchowdhuri@gmail.com (I.C.); 5 Department of Environmental Sciences, Faculty in Natural Resources and Marine Science, Tarbiat Modares University, Tehran 14115-111, Iran; zhalehsiabi@modares.ac.ir; 6 Department of Natural Engineering, Faculty of Natural Resources and Earth Science, Shahrekord Unversity, Shahrekord 8818634141, Iran; a.norouzi@stu.sku.ac.ir; 7 Department of Earth and Environment, AHC-5-390, Florida International University, 11200 SW 8th Street, Miami, FL 33199, USA; melessea@fiu.edu; 8 Faculty of Civil Engineering, Institute of Structural Mechanics, Bauhaus-Universität Weimar, 99423 Weimar, Germany; manouchehr.shokri@uni-weimar.de; 9 Environmental Quality, Atmospheric Science and Climate Change Research Group, Ton Duc Thang University, Ho Chi Minh City, Vietnam; amirhosein.mosavi@tdtu.edu.vn; 10 Faculty of Environment and Labour Safety, Ton Duc Thang University, Ho Chi Minh City, Vietnam

**Keywords:** artificial intelligence, groundwater, nitrate concentration, hydrology, groundwater contamination, environmental pollution, artificial neural network, deep learning, data science, machine learning, big data, agricultural contamination, agricultural pollution, prediction, hydrological model

## Abstract

Prediction of the groundwater nitrate concentration is of utmost importance for pollution control and water resource management. This research aims to model the spatial groundwater nitrate concentration in the Marvdasht watershed, Iran, based on several artificial intelligence methods of support vector machine (SVM), Cubist, random forest (RF), and Bayesian artificial neural network (Baysia-ANN) machine learning models. For this purpose, 11 independent variables affecting groundwater nitrate changes include elevation, slope, plan curvature, profile curvature, rainfall, piezometric depth, distance from the river, distance from residential, Sodium (Na), Potassium (K), and topographic wetness index (TWI) in the study area were prepared. Nitrate levels were also measured in 67 wells and used as a dependent variable for modeling. Data were divided into two categories of training (70%) and testing (30%) for modeling. The evaluation criteria coefficient of determination (R^2^), mean absolute error (MAE), root mean square error (RMSE), and Nash–Sutcliffe efficiency (NSE) were used to evaluate the performance of the models used. The results of modeling the susceptibility of groundwater nitrate concentration showed that the RF (R^2^ = 0.89, RMSE = 4.24, NSE = 0.87) model is better than the other Cubist (R^2^ = 0.87, RMSE = 5.18, NSE = 0.81), SVM (R^2^ = 0.74, RMSE = 6.07, NSE = 0.74), Bayesian-ANN (R^2^ = 0.79, RMSE = 5.91, NSE = 0.75) models. The results of groundwater nitrate concentration zoning in the study area showed that the northern parts of the case study have the highest amount of nitrate, which is higher in these agricultural areas than in other areas. The most important cause of nitrate pollution in these areas is agriculture activities and the use of groundwater to irrigate these crops and the wells close to agricultural areas, which has led to the indiscriminate use of chemical fertilizers by irrigation or rainwater of these fertilizers is washed and penetrates groundwater and pollutes the aquifer.

## 1. Introduction

Groundwater is among the essential freshwater resources for urban consumption, industries, and agriculture in the arid and semi-arid regions [1,2,3]. Increasing population, climate change, and over-abstraction of groundwater for irrigation could have considerable impacts on groundwater. The reasonable management of groundwater quantity and quality is a crucial issue that needs to be reviewed. Hence, to determine the sustainable management of groundwater, the evaluation of connected pressure at the different scales are vigorously essential [4,5]. Nitrate (NO_3_-) is the high pollutant in groundwater [6,7]; furthermore, NO_3_-concentration growth continues, with amplification of agricultural operations owing to the overuse of nitrogen fertilizers [8,9,10], manure management, and crop cultivation practices that move into the farming field [11,12]. Accordingly, the consumption of water polluted through nitrate can be connected to health problems, for example, cancers in adults via drinking water and skin contact [13,14]. For this purpose, groundwater-pollution predicting could assist managers of water resources and environmental protection in their probes to hamper groundwater pollution and to enhance its quality [15,16,17].

Several different machine-learning methods such as random forest (RF), support vector machine (SVM), artificial neural network (ANN) have been investigated to evaluate groundwater nitrate concentration susceptibility predictions [18,19]. The results of most of these studies showed that the best model to justify nitrate changes varied in each region. For instance, the BRT model in Nolan et al. [20], SVM in Sajedi-Hosseini et al. [21], ensured the maximum likelihood-based linear model in [22] performed better. In general, tree-based models showed high efficiency in various studies in other parts of the world, and most studies in this field have been done using these models. The RF model is strong to outliers and uncomplicated to exert in comparison to other data mining methods; it has the peculiarity to characterize the significance of each explanatory variable in the prediction outcome. Further, the RF model can access satisfaction results in comparison to the multivariate statistics or other machine learning methods such as SVM and ANN (due to local minima and overfitting problems) [22,23,24,25,26]. However, it does not compute regression coefficients or confidence intervals and acts as a black box because the individual trees could not be inquired one by one [27]. Based on the above issues, in the current research, other machine learning approaches such as Cubist regression (CB), Bayesian artificial neural network (Bayesian ANN) to overcome the above techniques were used. CB is a set of rules related to sets of multivariate methods [28] which do not recapture one final model like RF. The fact is that a particular set of predictor variables will select an actual prediction model depending on the rule that best fits the predictors [29]. Although Noi et al. [30] stated that Cubist regression and random forest algorithms have a good performance in estimating daily air surface temperature from dynamic combinations of MODIS LST data, Bayesian ANN notes to developing standard networks with posterior inference to regard a probability distribution of weights instead of a single set of weights [31]. Sahoo et al. [32] put forward Bayesian methods for water quality assessment and presented that the quality of water was improved during dry seasons more than during wet seasons owing to the dilution of pollutants [33,34,35,36].

According to the mentioned contents and studies, it can be said that optimal modeling and mapping of nitrate concentration in groundwater is important and vital to make efficient decisions in groundwater management. The sensitivity of groundwater studies in arid and semi-arid regions is more important and necessary due to the lack of access to sufficient surface water resources, and therefore, maximum using pressure is on groundwater resources. In previous studies, researchers used different machine learning structures such as decision trees and regression to model water pollution. In this study, in addition to the well-known machine learning structures, including decision tree and regression, we used the Bayesian framework for the first time to model and prediction of nitrate concentration in groundwater. Therefore, in nitrate studies of such areas, the use of different and new models and comparing the efficiency of these models to model and accurately map nitrate pollution is much more important than other areas. In the present study, for the first time, four modeling techniques, including Cubist, random forest (RF), support vector machine (SVM), and Bayesian artificial neural network (Bayesian ANN) were used to efficiency comparison of nitrate modeling in the Marvdasht watershed, Fars province, Iran. For this purpose, the data of nitrate concentration obtained from the Department of Water Resources Management (IDWRM) at 67 wells, as well as data of 11 important variables in the spatial distribution of nitrogen, including altitude, slope, plan curvature, profile curvature, rainfall, Piezometric depth, distance from residential, distance from river, K, Na and topographic wetness index (TWI) were used in June 2018.

## 2. Materials and Methods

### 2.1. Description of the Study Area

The Marvdasht watershed is one of the watersheds of Tashk-Bakhtegan and Maharloo lakes in Fars province. The basin is formed between 29°18′ to 30°22′ east longitude and 52°18′ to 53°40′ north latitude. The study area of the Marvdasht watershed is 3941 square kilometers at its widest and is the most complex watershed study area of Tashk-Bakhtegan and Maharloo lakes. The average long-term annual rainfall in the region is about 427 mm. Geologically the Marvdasht area of Kharameh has wide alluvial plains with mild slope and low slope, with deep to semi-deep soil with high fertility, and sediment thickness in this area sometimes reaches 200 m. The quality of water in these alluviums is suitable for the cultivation of all kinds of crops, and for this reason, a large area of land has been cultivated in rained and irrigated crops. The agricultural lands in the study area are devoted to the cultivation of cereals, rice, forage crops, sugar beet, vegetables, pesticides, citrus fruits, legumes, cotton, and oilseeds, of these, the largest area under cultivation is cereals (wheat and barley).

**Figure 1:**
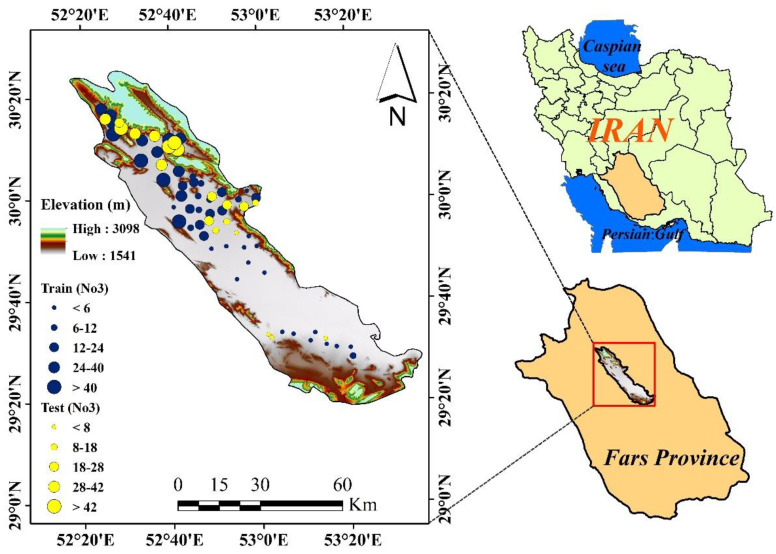
Location of the Marvdash watershed in Fars province, Iran.

Groundwater-nitrate concentrations were provided by the Iranian Department of Water Resources Management (IDWRM) at 67 wells during June 2018 (Figure 1). The highest nitrate value (56.74 mg/L) was in the northern parts of the watershed in agricultural soils with weak slopes. In the southern part of the basin, the concentration of nitrate was less than 6 mg/L, with the lowest level being 2.23 mg/L (Table 1). Various influential geo-environmental variables on nitrate concentration were assembled for the case study: elevation (m), slope (%), plan curvature, profile curvature, annual rainfall (mm), piezometric depth (m), distance from residential (m), distance from the river (m), (Sodium) Na (mg/L), (Potassium) K (mg/L), and TWI.

### 2.2. Methodology

**Figure 2:**
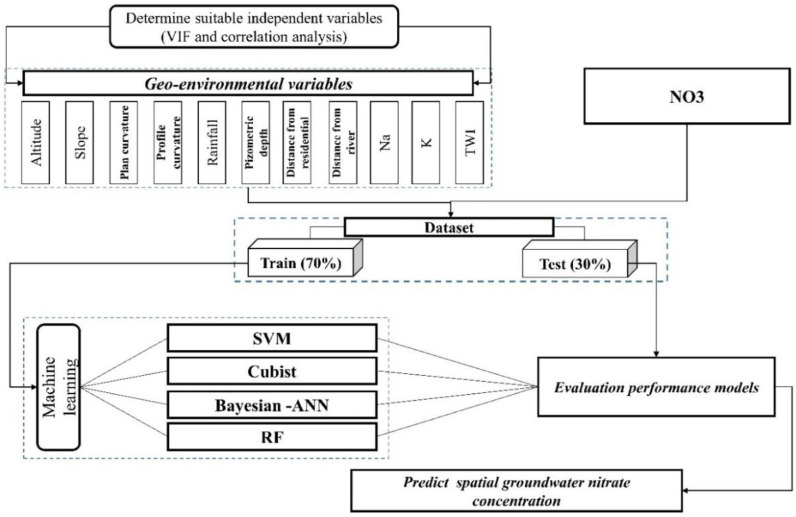
Methodological flow chart.

The overview of the nitrate concentration modeling relevant to VIF, Cubist, SVM, RF, and BNN has been summarized in a flowchart presented in Figure 2.

### 2.3. Dataset Preparation

**Figure 3:**
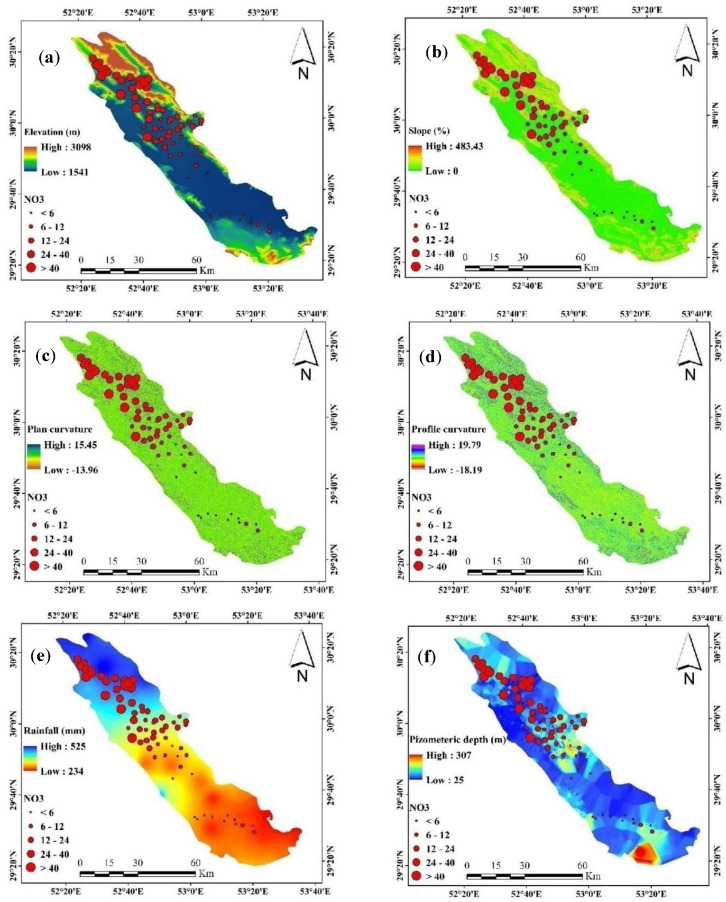

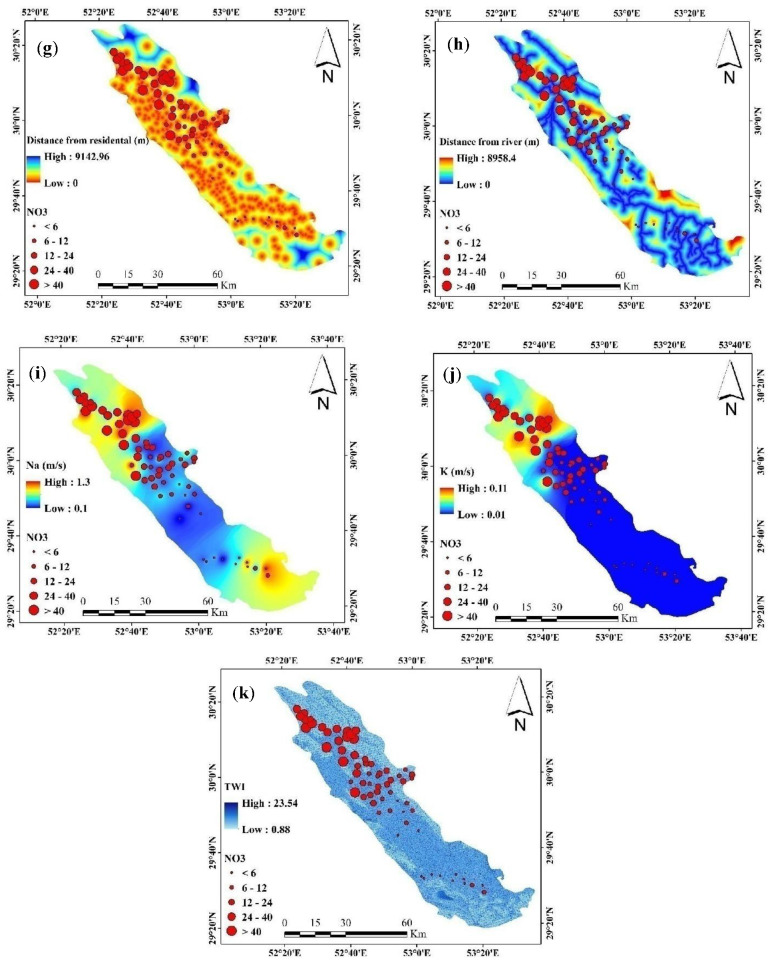
Groundwater vulnerability to NO_3_ factors: (**a**), elevation; (**b**), slope; (**c**), plan curvature; (**d**), profile curvature; (**e**), rainfall; (**f**), piezometric depth; (**g**), distance from the river; (**h**), distance from residential; (**i**), Sodium (Na); (**j**), Potassium (K); (**k**), TWI.

In this study, 11 possible influential factors connected to the innate and specific groundwater vulnerability to NO_3_ were applied including elevation, slope, plan curvature, profile curvature, distance from river, distance from residential, piezometric depth, rainfall, Na, K, and topographic wetness index (TWI) (Figure 3).

The DEM map was obtained with a pixel size of 12.5 m from the ALOSPALSAR sensor, the slope map, plan curvature, profile curvature in the GIS software environment were prepared based on DEM. Slope is one of the effective factors in determining the penetration of pollution into the saturation zone. Plan curvature examines the maximum slope in a vertical side. It has illustrated the convergence and divergence of water flow in the ground surface that positive and negative values represent the divergence and convergence of water flow in the study area, respectively. Profile curvature is an equal condition to the maximum slope in a specific direction and calculated as the slope perpendicular to the slope gradient and has negative and positive values. As opposed to, negative and positive values in profile curvature display convexity (increasing flow velocity) and concavity (reducing flow velocity), respectively [37].

Groundwater movement is due to the current spatial distribution of piezometric levels that have varied severely over a period of years [38]. This variation was related to the overexploitation of groundwater resources for drinking, industrial, and agricultural uses [39]. Increases in piezometric levels cause relevant impacts of anthropogenic factors related to groundwater and ground deformations [40]. The piezometric level demonstrates whether the NO_3_-can promptly arrive at the groundwater-surface. The shallower water depth could high the probability of NO_3_-contamination [41].

Rainfall is a climate factor and can be assumed as the aquifer inputs that impact on groundwater contamination through water budget [42]. The rainfall flows to groundwater recharge, which engenders the leaching of soil NO_3_- [43]. The rainfall map of the constituency was prepared from the statistics of seven synoptic stations around the constituency with a statistical period of 27 years and based on the inverse distance weighted interpolation (IDW) interpolation method. Sodium and Potassium are different dissolved inorganic constituents that are naturally available in the water. There are permissible limits in most of the groundwater. The increasing sodium and potassium in the groundwater are presumably relevant to the influence of leaching of soaps and sites close to agriculture areas that utilize fertilizer and agricultural activities [44]. The Sodium and Potassium map was prepared based on the obtained amount of these elements in 62 studied wells using IDW interpolation method. The river is one of the factors of water exchange between the river and groundwater aquifers, and most water exchanges take place in the areas adjacent to the river. Distance from residential is a factor that draws potential nitrate pollution from the transfer of waste and wastewater. The map of the distance from the river and the distance from residential based on the Euclidean extension was obtained in GIS software. SAGA-GIS software was applied to map TWI. The TWI was estimated with the help of the following method:

(1) $$TWI=\;In\;\left(\frac{as}{tanB}\right)$$

where, as refers to the catchment area, and tanB represent slope angle [45].

### 2.4. VIF

The tolerance and variance inflation factors (VIF) are two indices that are applied generally for examining the multicollinearity of variables. Multicollinearity is a statistical evaluation tool indicating that one can be linearly predicted concerning the others with a non-trivial degree of accuracy [46]. It can be exerted to remove extremely correlated agents from the modeling process and to elude any terminated bias in models’ results. These indices are determined, as shown in Equations (2) and (3):

(2) $$Tolerance=1-R^{2}J$$

(3) $$VIF=\frac{1}{Tolerance}$$

where *R*^2^*J* demonstrates the determination of the regression coefficient in influential factors *j* on whole the other influential factors. A tolerance of >0.10 and variance inflation factors (VIF) > 5 illustrate a multicollinearity problem [47,48].

### 2.5. Machine Learning Methods

#### 2.5.1. Cubist

Cubist regression is a rule-based method that was created relevant to the incorporation of the Quinlan opinion. CB is presently a more commonly applied regression and classification method because it was carried in R by Kuhn et al. [49] in 2013. Conceptually, the Cubist regression method is the tree that expands, and the endpoint leaf entails a linear regression model for modeling. The Cubist model produces a set of “if-after-after” rules in which each rule has a connected multivariate linear model. The mentioned method is applied to compute the forecasted amount while the set of covariates persuades the rule conditions. CB a set of rules related to sets of multivariate methods that do not recapture one ultimate method, such as RF. The facts that a particular set of predictor factors will select a real prediction method depend on the rule that properly matches the predictors [27]. The Cubist type adds boosting with training consultants (commonly higher than one), which is related to the “boosting” algorithm by consecutively advancing groups of trees with modified weights [50].

#### 2.5.2. Support Vector Machine (SVM)

Support vector machine is a classification of discrimination monitoring or statistical theory-based model which was introduced by Vapnik [51] in the mid-1990s. SVM was developed to dissolve complicated classification and regression issues. SVM is a method for estimating a function that is estimated to a real number based on training data from an input object. In regression problems, input vectors are mapped to a multidimensional space; a hyperplane is then created that separates the input vectors as far apart as possible. A kernel function is used to solve the problem of performing operations in large dimensions. In fact, using the kernel function, the problem of multidimensional and nonlinear calculating is solved [52,53].

#### 2.5.3. Random Forest (RF)

Random forest (RF) is a popular supervised machine learning method for modeling various phenomena [18,54,55] and is effective for data prediction and explanation purposes. RF can calculate an unbiased error evaluated by bootstrapping [56]. The dataset exerted for RF is separated into two parts that the first part is related to training and contains 70 percent of the dataset randomly selected with a replacement, and a validation subdataset containing the remaining 30 percent. RF demonstrates averaging multiple decision trees, trained on various portions of the same training data set, to reduce the prediction variance [57]. The trees in RF expand to the largest range feasible without pruning, and they are combined by averaging trees. For calculating variable importance and assessing an unbiased calculate of the test set error was applied out-of-bag (OOB) samples. There is no need for cross-validation of OOB samples [18].

#### 2.5.4. Bayesian Artificial Neural Network (Bayesian ANN)

A Bayesian neural network is a neural network with a former distribution on its weights [29]. In other words, it notes developing standard networks with posterior inference to consider a probability distribution of weights instead of a single set of weights. In the Bayesian framework, uncertainty relevant to the relationship between inputs and outputs is originally attended through an assumed former distribution of parameters (weights and biases). This former distribution is renovated to posterior distribution using a likelihood function subsequent Bayes’ theorem while data are observed. This posterior distribution is entitled to the objective function of a network in the Bayesian learning approach [58].

### 2.6. Validation and Accuracy Assessment

The four models, namely the best-fit goodness or coefficient of determination (R^2^), minimal absolute error (RMSE and MAE), and model efficiency (NSE) measurements, were accurately evaluated to specify the most impressive approach. The coefficient of determination (R^2^) indicates the coefficient of variance explanation or dependent variable variation by a set of independent variables. The value of this coefficient fluctuates between zero and one. The closer the value of this coefficient is to one, it indicates that the independent variables have been able to predict a large amount of variance or the behavior of the dependent variable, and the closer this value is to zero, the less explanation this variable [59,60].

Nash–Sutcliffe efficiency (NSE): The Nash–Sutcliffe efficiency (NSE) is a normalized statistic that characterizes the relative extent of the residual variance (“noise”) contrasted to the calculated data variance (“information”) [61]. NSE demonstrates how well the plot of observed versus simulated data fits the 1:1 line. NSE ranges between −∞ and 1.0 that NSE = 1 is the optimal value.

RMSE is one of the extensively applied error-index statistics [62]. It is commonly admitted that when the lower the RMSE, the model efficiency is improved. It qualifies what is regarded as a low RMSE based on the observation’s standard deviation [63]. Furthermore, mean absolute error (MAE) is another error-index that is frequently used in model evaluation. The value of 0 demonstrates a complete fit. RMSE and MAE values of less than half the standard deviation of the calculated data can be regarded low and that either is suitable for model assessment.

(4) $$MAE=\frac{\sum_{i=1}^{n}\left(N_{o}-N_{p}\right)}{n}.$$

(5) $$RMSE=\;\sqrt{\frac{1}{n}{\left(N_{o}-N_{p}\right)}^{2}}.$$

(6) $$R^{2}=\frac{\sum_{i=1}^{n}\;(N_{o}-\bar{N_{o}})*(N_{p}-\bar{N_{p}})}{{\left(\sum_{i=1}^{n}{\left(N_{o}-\bar{N_{o}}\right)}^{2}\right)}^{0.5}*{\left(\sum_{i=1}^{n}{\left(N_{o}-N_{p}\right)}^{2}\right)}^{0.5}}$$

(7) $$NSE=1-\frac{\sum_{i=1}^{n}{\left(N_{o}-N_{p}\right)}^{2}}{\sum_{i=1}^{n}{\left(N_{o}-\bar{N_{o}}\right)}^{2}}.$$

where $N_{o}$ is the observed value of dependent variables, $N_{p}$ is the estimated value of dependent variables, and ${\bar{N}}_{o}$ is the observed mean value of dependent variables.

## 3. Results

### 3.1. Exploratory Data Analysis and Data Statistic Analysis

**Figure 4:**
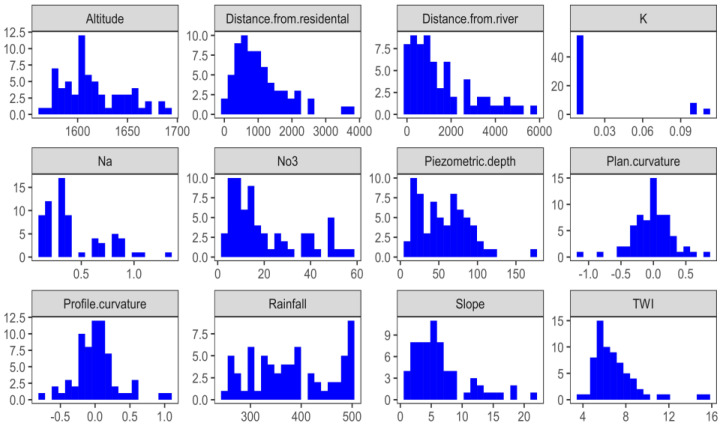
Exploratory data analyses.

A total of eleven potential exploratory variables for groundwater nitrate concentrations were examined in this study (Figure 4). The first variable, the altitude of this Marvdasht watershed, varies from 1541 to 3098 m above mean sea level, but most of the altitude in this area is between 1550 and 1700 m. Approximately 12.5 percent of this study area is located at an altitude of 1600 m. Topographical elevation has a significant impact on nitrate concentrations in groundwater. The lowest elevation with flat topography has a relatively high concentration of nitrate compared to a high elevation with a steep topography [64]. The slope ranges from 1 to 20 percent in this watershed, where 5 percent of the slope has a larger pixel area. Generally, flat slopes and flat land are mostly associated with nitrate in groundwater, but steep slopes at high altitudes have a major impact on nitrogen loss due to the large surface runoff, resulting in minute nitrate leaching into groundwater [65]. Low land and low slopes are closely linked to agricultural land, which is why this type of topography causes nitrate concentrations in groundwater.

Residential areas are a significant source of nitrate concentrations in groundwater, such as inorganic and organic fertilizers, concentrated animal feed operations (CAFOs), sewage, sewer leakage, and septic systems [66]. In this study, the main sources of groundwater nitrate from the residential area are below 2000 m of the buffer. Nitrate leakage from the flood plain is the main source of mineral contamination in the natural aquifer, where the process is accelerated by agricultural drainage [67,68].

The distance to the river varies from 0 to 8958. 4 m, but the main part is located between 0 to 6000m. The Marvdasht watershed, potassium (K), and nitrate both contaminate the groundwater and there is a positive relationship between the two minerals because they are used as fertilizers [69]. This area, below 0.3 K concentrations, has the highest concentration area. Sodium (Na) is also related to mineral contamination in groundwater and is closely associated with nitrates from irrigation and precipitation leaching through soils [70]. Sodium in groundwater below 0.5–1.0 mg/L is generally available here.

Aquifer nitrate concentrations are mostly observed at shallow Piezometric depth or water table depth, and the average Piezometric depth in this study area varies from 0 to 125 m [71]. The plan curvature and the profile curvature mainly from −0.5 to 0.5 in this watershed, but a high percentage of the areas do not have a curvature or a flat area. Rainfall is a climate factor of groundwater nitrate concentration; high average rainfall dilutes nitrate in soil and further increases the process of leaching [11].

The average annual rainfall ranges from about 300 to 500 mm, and the high percentage of the study area is over 500 mm. The hydrological status of the topography is measured by the TWI, which determined the pattern of mineral contamination in groundwater. Most of the area of this watershed belongs to low to medium humidity in the topography. The response is the nitrate concentration that is spatially predicted by the eleven predictors, and the nitrate (NO_3_) data observed ranges from 1 to 58 mg/L but most of the NO_3_ data ranges from 1 to 20 mg/L.

The results of the statistical characteristics of the independent variables and the dependent variable in the two stages of training and testing are shown in Table 2.

### 3.2. Correlation Analysis

**Figure 5:**
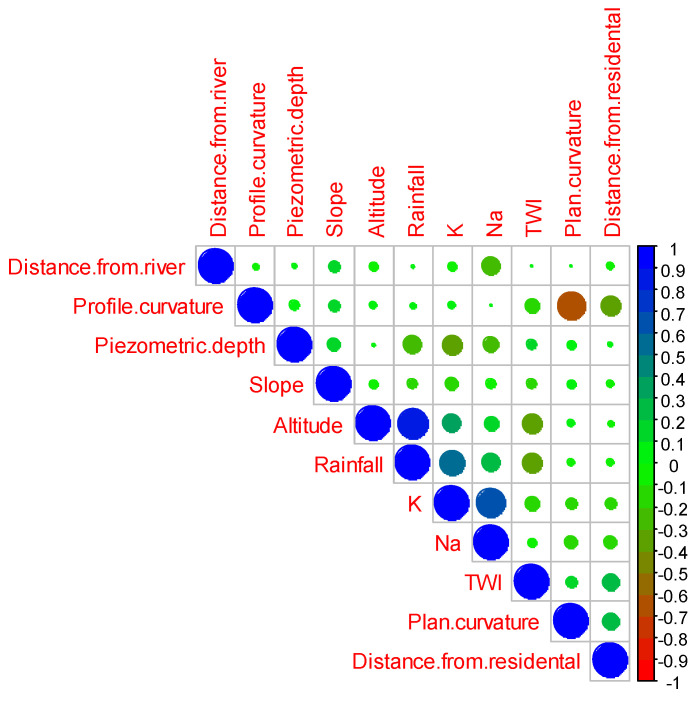
Correlation analyses parameters based on Spearman.

The Spearman correlation matrix shown in Figure 5 shows the monotonic relationship between aquifer nitrate concentration potential variables. The correlation matrix shows that the four have a strong relationship, i.e., altitude is strongly correlated with precipitation, K is positively correlated with Na, and precipitation is positively correlated with K. On the other hand, the curvature of the plane is negatively correlated with the curvature of the profile. TWI is moderately correlated with altitude and precipitation. Piezometric depth is moderately negatively related to K but positively related to precipitation. The other contamination of the aquifer, Na, is moderately positively correlated with the distance from the river. The rest of the interrelationships have a low to a medium positive relationship, and some have a negative relationship.

### 3.3. Multi-Collinearity Analysis

Sometimes more than two variables are involved in a linear relationship, and the data have a problem that can be reliably linked to the difficulty of estimating the model parameter, called multicollinearity [72]. Tolerance (TOL) and inflation factor variance (VIF) are two key indicators for the evaluation of multicollinearity between variables. If the TOL value is more than 0.2 and the VIF value is greater than 10, there is no multicollinearity, but if the independent variable does not comply with the above-mentioned rules, there is a multicollinearity between them [73]. The TOL and VIF values in this study are calculated and shown in Table 3, showing that there is no multicollinearity between any of the variables considered in this groundwater nitrate susceptibility assessment.

### 3.4. Validation of the Models

**Figure 6:**
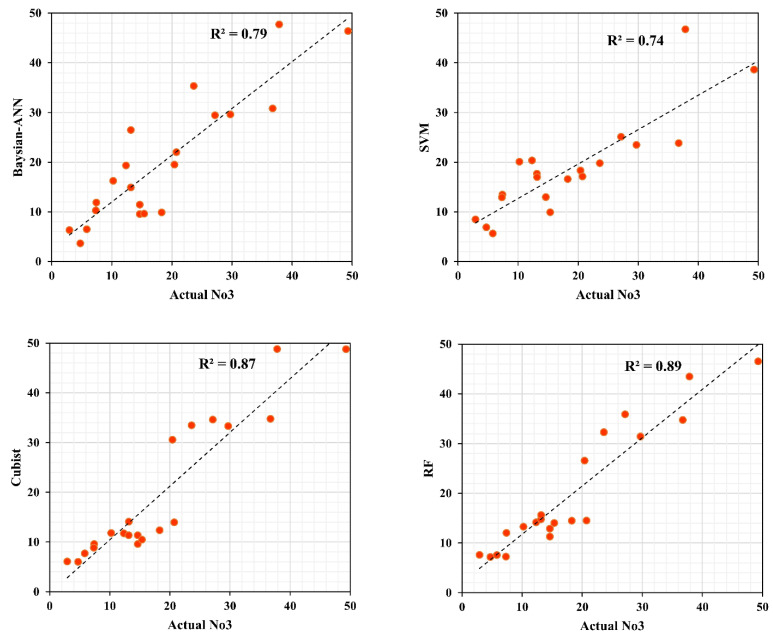
Scatter plot Bayesian ANN, SVM, cubist, and RF models for groundwater nitrate concentration in the validation stage.

**Figure 7:**
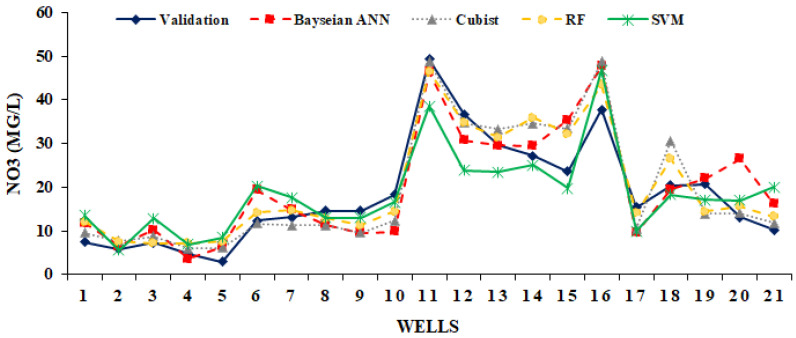
Result of Bayesian ANN, SVM, cubist, and RF models for groundwater nitrate concentration in the validation stage.

This section describes the model performance associated with the model results in both the training and validation phases of the model. The expected result of the groundwater nitrate concentration was evaluated based on well nitrate data. In the training phase, 70 percent of the data was used to train the predictive model and 30 percent of the data was used to test the predictive model. The coefficient of determination (R^2^), root mean square error (RMSE) and mean absolute error (MAE) and Nash–Sutcliffe efficiency (NSE) measurements for four models in the training and testing phase have been summarized in Table 4. All the evolution results indicate that the Cubist, RF, SVM, and Bayesian ANN machine learning models have a good performance and a sufficient data span for the training and testing process. The assessment result of the models found the best performance by the Cubist model because it has the highest R^2^ (0.96) and NSE (0.95) and the lowest absolute error (RMSE, 3.52 and MAE, 2.52). Based on R^2^, RMSE, MAE, and NSE, Cubist models RF, SVM, and Bayesian ANN have improved their performance in groundwater nitrate modeling potential. Furthermore, in the case of the test phase (using the validation dataset), the prioritization result also showed the best performance similar to the training phase. However, the RF model (R^2^, 0.89; RMSE, 4.24; MAE, 3.55; NSE, 0.87) is capable of showing the best results compared to the Cubist and the other three models. Subsequently, the Cubist, Bayesian ANN, and SVM models have a good test performance. Predictive groundwater nitrate concentrations and actual nitrate concentrations from 21 wells (30 percent well) were compared based on the scatter plot in Figure 6 and continuous profile chart Figure 7. All models listed have more or less the same scenario, with nitrate data validation points.

**Figure 8:**
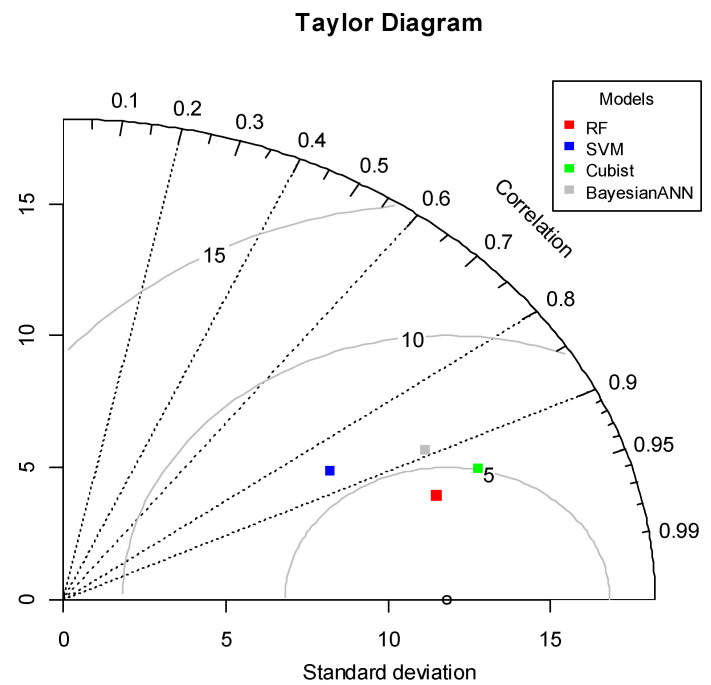
Taylor diagram of observed and simulated groundwater nitrate concentration susceptibility values by Cubist, SVM, RF, and Bayesian ANN models.

Figure 8 illustrates a two-dimensional graphical presentation of observed and simulated groundwater nitrate concentrations for the Cubist, RF, SVM, and Bayesian ANN models, called the Taylor diagram. This diagram is one of the graphical presentations used to assess the accuracy of the forecast based on a number of statistical indicators [74]. Statistical indicators such as correlation coefficients, standard deviations, and root mean square error for predictive groundwater nitrate concentrations have been measured. In this study, the Taylor diagram provides a spectacular overview of the relationship between the predicted and observed groundwater nitrates in the Marvdasht watershed. And all predictive models have slightly similar performance in nitrate prediction (Figure 8). However, the proposed Cubist model indicates that the concentrations of nitrate in groundwater are most closely coordinated.

### 3.5. Spatial Groundwater Nitrate Susceptibility

**Figure 9:**
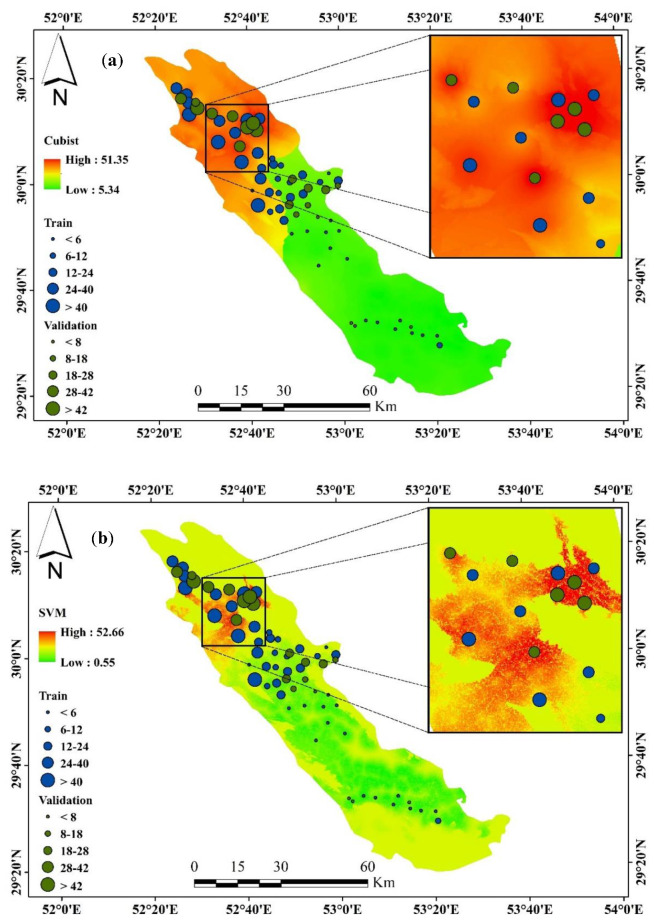

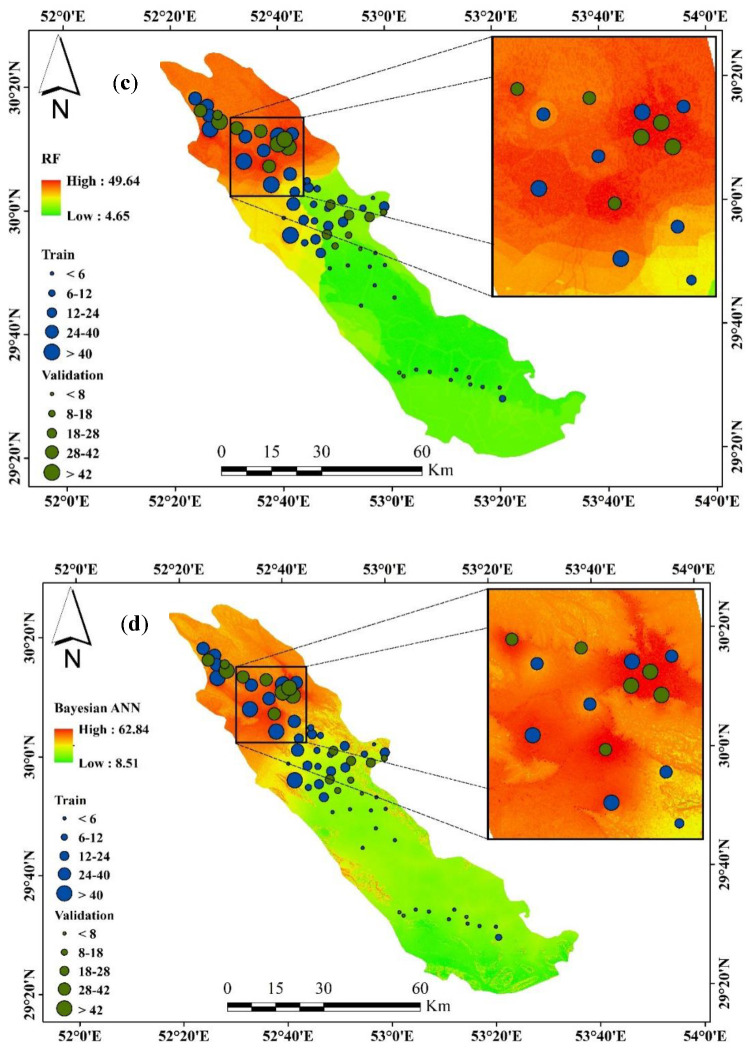
Spatial groundwater nitrate concentration susceptibility using (**a**) Cubist, (**b**) SVM, (**c**) RF, and (**d**) Bayesian ANN models.

Groundwater nitrate concentration susceptibility maps were produced using four machine learning methods. In all models, the nitrate susceptibility maps were shown with the same symbol in Figure 9. Cubist groundwater susceptibility map ranges from 5.34 to 51.35 mg/L, SVM ranges from 0.55 to 52.66 mg/L, RF ranges from 4.65 to 49.64, and Bayesian ANN model susceptibility maps range from 8.51 to 62.84 mg/L. The northwestern part of the study area is a high concentration of groundwater nitrate, the main findings of all susceptibility maps. The southern portion of this watershed has a low nitrate concentration area. The cubist model showed that high groundwater nitrate contamination was higher than the other maps, and the SVM model demonstrated that low groundwater nitrate contamination was higher than the other models.

### 3.6. Importance Value

**Figure 10:**
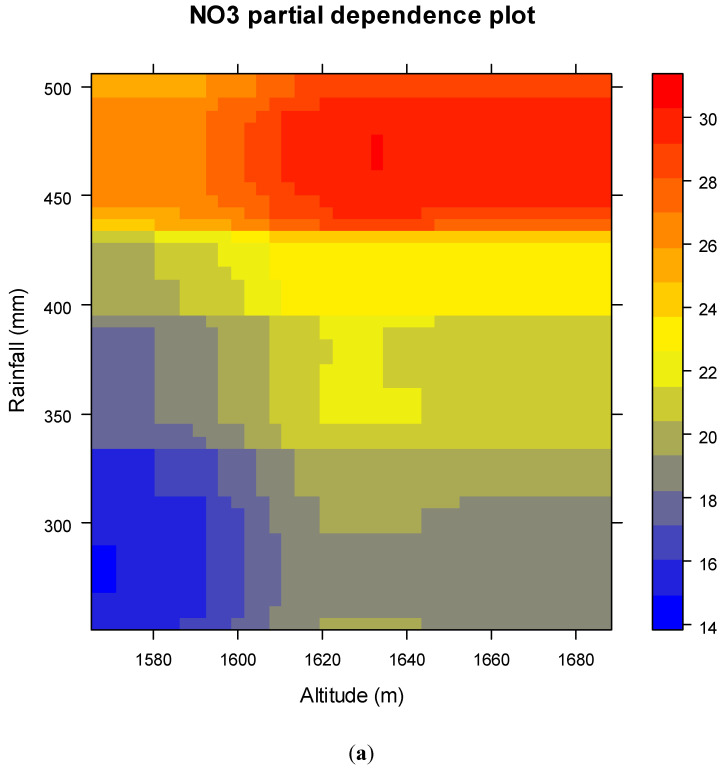

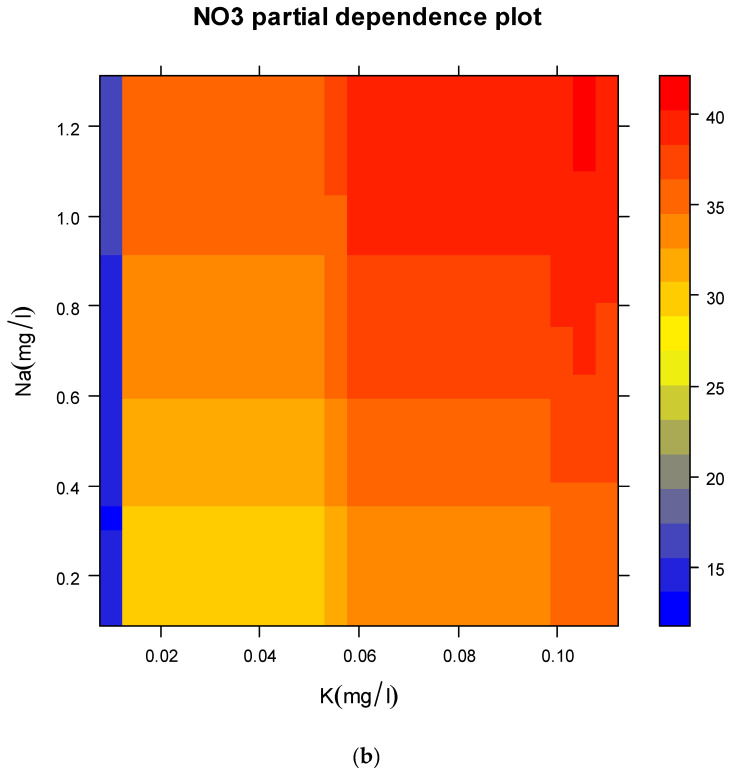
NO_3_ partial dependence plot for importance variable: (**a**) Altitude and Rainfall, (**b**) K, and Na.

The assessment of the significant variable result based on the mean decrease of the Gini-coefficient using the RF model is shown in Table 5. Moreover, the important result shows the all the determine factors generally contribute to nitrate contamination in groundwater and groundwater nitrate susceptibility. However, altitude, rainfall, and K are the most important factors, followed by distance to a river, distance from residential TWI, and Na, respectively. The importance value of the above result also showed the strongest relationship between altitude, distance to the river, distance from residential, rainfall, K, Na, and piezometric depth, and the groundwater nitrate contamination. However, these association results indicated that the majority of nitrate contamination occurred in high elevations and rainfall near rivers and residential and low piezometric depth regions. On the other hand, the curvature of the plan, the curvature of the profile, the TWI, and the slope are of low importance for nitrate contamination of groundwater. If we see a partial dependence plot, high altitudes and severe rainfall are the main cause of excess nitrate concentrations in groundwater. High soil contamination of K and Na minerals may be concentrated on nitrates in groundwater (Figure 10).

## 4. Discussion

Determination of groundwater nitrate concentration causative factors (GNCfs), generation of groundwater nitrate concentration susceptibility (GNCSMs), and selection of the best-fit model are the early stages of groundwater nitrate concentration hazard, and the current research has been successful. The final maps of groundwater nitrate contamination susceptibility show the less diversity of the four modern machine learning models. The comparison between the susceptibility map and the nitrate distribution shows a clear link between the level of nitrate concentrations observed and the level of susceptibility observed. The highest area is the limited probability of nitrate concentration (Figure 8), despite the moderate to high susceptibility in the Marvdasht watershed. When we talk about the groundwater susceptibility model, different statistical and empirical models for predicting groundwater mineral concentrations have been reviewed over the last decades [75,76,77]. However, these susceptibility models have some limitations and assumptions, and recently data mining with machine learning approaches has been effectively popularized due to their ability to analyze the multifarious relationship between predictors and response [34,78]. Alongside this, a number of different machine learning models, along with a different statistical model, have been successfully applied [79,80]. This work was carried out through four data mining and machine learning approaches to a comparative discussion of the GNCSM. In addition, several researchers used R^2^, RMSE, MAE, and NSE to assess the predictive capability of these models [81]. Each modeling approach was evaluated, taking into account both the nitrate concentration training and the nitrate concentration test or the validation subgroups, using the reliability measures referred to above. Based on the results of the R^2^, NSE, MAE, and RMSE training data sets, the Cubist model had the best performance, followed by the RF, RF, SVM, and Bayesian ANN models, but the RF model had the best reliability during the test phase (Table 4), Rahmati et al. [34] also showed that the RF model is better than the two models KNN and SVM in predicting the concentration of nitrate in groundwater. In addition, Ouedraogo et al. [82] in nitrate concentration modeling using RF and MLR showed that the RF has a better performance than MLR. The RF model is a combination of a set of decision trees to which a subset of data is injected. Each of the algorithms performs a learning operation that predicts a result when predicting, that is, when a new set of data is given to the algorithm for prediction, each of which is learned. Finally, the RF algorithm can use voting to select the decision tree that received the most votes and use it as the final output to perform the modeling operation, therefore, this model can provide good performance in simulating various phenomena [18,54].

According to the results of importance value altitude, rainfall, K and Na had the highest importance in groundwater nitrate concentration mapping. Similarly, Honarbakhsh et al. [83] showed that the conditioning factors such as Mg2+, Na+, K+, and total hardness affect the groundwater quality index (GWQI) in this study region. Important variables of groundwater nitrate concentrate susceptibility mapping are significantly affected by the methods used and the characteristics of the study area [34]. According to the important parameters result and the partial dependence plot (Figure 9) for the importance variable, there was a direct relation between altitude, rainfall, K and Na with nitrate (NO_3_) concentration in groundwater that means increasing the degree above factors may increase the nitrate in groundwater. However, the results of the study will help the planners for the management of groundwater for a different purpose.

According to the results, the concentration of nitrate is higher in the northern regions of the basin, which is higher in these agricultural areas than in other areas. The most important cause of nitrate pollution in these areas is activities such as rice, summer, and cereals in these areas and the use of groundwater to irrigate these crops and the wells close to agricultural areas, which has led to the indiscriminate use of chemical fertilizers by irrigation or rainwater of these fertilizers is washed and penetrates groundwater and pollutes the aquifer. Tian et al. [84] and Nejatijahromi et al. [85] also showed that the use of chemical fertilizers is one of the sources of groundwater pollution based on nitrate.

## 5. Conclusions

Nitrate is one of the pollutants of groundwater resources. In recent years, owing to agricultural development and human activities, their average amount in groundwater is increasing. The solution of natural sediments containing nitrate in water, plant decomposition, animal waste, municipal waste, and domestic and industrial wastewater, and the use of nitrogen fertilizers are among the sources of nitrate entering surface and groundwater. In this study, the potential of machine learning models including SVM, cubist, RF, and Bayesian-ANN in predicting pollution of nitrate concentration in groundwater by agriculture activities of the Marvdasht plain of Fars Province, was investigated. The results of ML models showed these models are capable of predicting nitrate pollution in groundwater. RF model with NSE = 0.87 is capable of showing the best results compared to the other three models. The assessment of the significant variable result based on the mean decrease of the Gini-coefficient using the RF model showed altitude, rainfall, and K are the most important factors in nitrate pollution modeling. The results of nitrate contamination zoning showed that the northern parts of the watershed, which include the upstream areas of the watershed, have more nitrate contamination compared to the southern parts of the watershed. Unfortunately, in recent years, due to the lack of awareness and mismanagement of wastewater, most farmers in the upward Marvdasht watershed irrigate their meadows through sewage collected during the solitary hours, especially at night, which this, along with the use of chemical fertilizers, makes groundwater resources more polluted. Due to the fact that downstream farmers use groundwater for drinking and agriculture, water pollution puts their health at risk. Regular monitoring of groundwater over a period of time and informing farmers in the area about the use of unconventional water and chemical fertilizers could help manage and prevent excessive pollution of these water resources.

## Figures and Tables

**Table 1:** Descriptive statistics of nitrate concentration.

Number of Wells	Mean	Minimum	Maximum	Standard Deviation
67	20.029	2.23	56.74	15.50

**Table 2:** The results of the statistical characteristics in the two stages of training and testing.

Variables	Train Data				Test Data			
	Mean	SD	Min	Max	Mean	SD	Min	Max
Altitude (m)	1616.13	33.28	1568.00	1694.00	1615.15	26.36	1567.00	1663.00
K (mg/lit)	0.03	0.04	0.01	0.11	0.02	0.03	0.01	0.10
Na (mg/lit)	0.44	0.29	0.10	1.30	0.39	0.25	0.10	1.10
Plan curvature	−0.03	0.30	−1.13	0.64	−0.02	0.35	−0.85	0.82
Profile curvature	0.05	0.31	−0.58	1.09	−0.06	0.31	−0.73	0.56
Pizometric depth (m)	55.04	33.95	12.58	171.04	57.72	29.31	6.84	110.34
Rainfall (mm)	381.97	81.26	254.10	503.02	383.03	73.45	267.03	498.84
Distance from residential (m)	1025.03	710.90	30.00	3777.74	956.73	675.58	42.43	3606.24
Distance from river (m)	1568.35	1399.89	0.00	5193.12	1559.50	1555.95	84.85	5730.08
Slope (%)	6.36	4.27	1.32	21.29	6.91	4.92	1.32	18.49
TWI	6.90	2.40	3.84	15.64	6.95	1.30	4.75	9.69
NO_3_ (mg/lit)	20.99	16.62	2.23	56.74	18.23	12.23	4.82	49.83

**Table 3:** Analyses of variables multi-collinearity.

Row	Variables	VIF	Tolerance
1	Altitude	3.72	0.27
2	Slope	1.12	0.89
3	Plan curvature	1.95	0.51
4	Profile curvature	2.01	0.49
5	Rainfall	4.44	0.22
6	Piezometric depth	1.39	0.72
7	Distance from residential	1.18	0.84
8	Distance from river	1.22	0.82
9	K	2.58	0.39
10	Na	2.24	0.45
11	TWI	1.25	0.67

**Table 4:** The predictive capability of head gully erosion models using train and test dataset.

Models	Stage	Parameters			
		R^2^	RMSE	MAE	NSE
Cubist	Training	0.96	3.52	2.52	0.95
	Validation	0.87	5.18	4.06	0.81
SVM	Training	0.94	4.24	2.73	0.94
	Validation	0.74	6.07	5.07	0.74
RF	Training	0.96	3.66	2.72	0.95
	Validation	0.89	4.24	3.55	0.87
Bayesian ANN	Training	0.88	5.89	4.56	0.88
	Validation	0.79	5.91	4.67	0.75

**Table 5:** Importance value.

Row	Variables	Importance Value
1	Altitude	2.35
2	Slope	0.91
3	Plan curvature	0.74
4	Profile curvature	0.67
5	Rainfall	3.15
6	Piezometric depth	1.09
7	Distance from residential	0.86
8	Distance from river	0.98
9	K	6.09
10	Na	1.84
11	TWI	1.01
